# Unravelling the community structure of the climate system by using lags and symbolic time-series analysis

**DOI:** 10.1038/srep29804

**Published:** 2016-07-11

**Authors:** Giulio Tirabassi, Cristina Masoller

**Affiliations:** 1 Departament de Fisica, Universitat Politecnica de Catalunya, Colom 11, ES-08222 Terrassa, Barcelona, Spain

## Abstract

Many natural systems can be represented by complex networks of dynamical units with modular structure in the form of communities of densely interconnected nodes. Unraveling this community structure from observed data requires the development of appropriate tools, particularly when the nodes are embedded in a regular space grid and the datasets are short and noisy. Here we propose two methods to identify communities, and validate them with the analysis of climate datasets recorded at a regular grid of geographical locations covering the Earth surface. By identifying mutual lags among time-series recorded at different grid points, and by applying symbolic time-series analysis, we are able to extract meaningful regional communities, which can be interpreted in terms of large-scale climate phenomena. The methods proposed here are valuable tools for the study of other systems represented by networks of dynamical units, allowing the identification of communities, through time-series analysis of the observed output signals.

Many real-world complex systems can be represented in terms of networks of interacting nodes embedded in space. Examples include power grids, fiber-optic networks, road networks, flight connections, etc^1^,^2^,^3^. Such networks are usually organized in modules or communities of densely interconnected nodes^4^,^5^,^6^,^7^,^8^,^9^,^10^,^11^. The spatial embedding of the network can hidden the underlying community structure, rendering the identification of communities a challenging a task^12^,^13^,^14^. The effects of space in the topology of the network are particularly important when the network is built with correlation analysis of output signals which are recorded at a regular grid of observation points. Examples of this situation include brain functional networks^15^,^16^,^17^ and climate networks^18^,^19^,^20^,^21^,^22^.

Here we focus on climate networks, which provide relevant insight into global climate phenomena^23^,^24^,^25^,^26^,^27^,^28^,^29^,^30^,^31^. Previous work has shown that climate communities reveal coherent subsystems^32^, can be used for model inter-comparisons^33^, and can advance climate predictability^34^. For example, communities obtained from the analysis of sea surface temperature (SST) reveal information about long-term SST variability^35^. In our approach, a climate community is understood as a set of geographical regions that share some common property (dynamical or statistical) of the climate in those regions. Therefore, our approach differs from (and is complementary to) that aimed at performing a dimensionality reduction. For example, the work by Runge *et al*.^36^ allows identifying geographical regions which are important for spreading and mediating perturbations. The methodology proposed in ref. 36 reduces a gridded data set to a set of principal components representing relevant subprocesses; then, a causal analysis is made to distinguish direct from indirect interactions. The reduction of the dataset to a set of principal components is done by detecting components that cannot be generated by a surrogate model^37^, and allows reducing the dimensionality of large climate datasets into spatially localised components. In contrast, the approach proposed here is not intended for dimension reduction, but rather, it is aimed at identifying geographical regions (not necessarily close) such that climate datasets in those regions have similar properties.

The existence of such regions is expected because of the physical processes that govern our climate (ocean and atmospheric processes, solar forcing, vegetation, human activity, etc.), which ultimately determine local climate variability. These processes act in a similar way in distant regions (having similar effects) and therefore, distant regions can have similar climate. Examples include tropical rainforests, dry and arid regions, maritime regions, etc. Given the complexity of climate phenomena, the community structure uncovered will depend on the property of the climate being analyzed, and the method used to construct the network should also be adapted to extract the relevant information from climate datasets.

Within the standard approach for constructing climate networks, the strength of the links is determined by correlation analysis (for example, by using the Pearson coefficient or the mutual information). Due to physical proximity (i.e., to the spatial embedding of the network), the nodes are linked mainly to neighboring nodes, while long distance links are rather scarce. Therefore, the standard way to construct a climate network does not allow detecting communities that represent distant regions which have similar climatic properties, because in these networks the northern and southern hemispheres are indirectly or only weakly connected. The spatial effect can hide, for example, the fact that distant extratropical land masses (in the two hemispheres) are likely to have similar climate.

Here propose and validate two methodologies to overcome this problem. From time-series recorded at a regular grid of points covering the Earth’s surface, the methods extract different and relevant properties of our climate. With the first method, which is based in computing mutual lags between time-series, we are able to infer communities defined by regions in which the oscillations of a climate variable (the surface air temperature, or the geopotential height) are in-phase; with the second method, which is based in symbolic time-series analysis, we group together regions that share similar properties of the symbolic dynamics. We validate these methods by uncovering meaningful communities, which can be related to known properties of the climate system.

## Data

We analyze monthly-averaged surface air temperature (SAT) and geopotential height (GH) reanalysis data from NCEP/NCAR (state-of-the-art model simulation with data assimilation using past observed data where and when is available^38^). The data covers the period from January 1948 to May 2012 (*T* = 773) data points and has a spatial grid resolution of 2.5 degrees (*N* = 10226 nodes). The data can be freely downloaded from the NCEP/NCAR reanalysis project webpage:

http://www.esrl.noaa.gov/psd/data/reanalysis/reanalysis.shtml

## Time Lags Method

The first method proposed for community identification unveils geographical regions in which the oscillations of a climate variable are in-phase, revealing similar response to annual solar forcing. To identify such regions, for each time-series we first compute the annual cycle, and then compare the mutual lags among all pairs of time-series. Our motivation to compute the lag time between the two seasonal cycles (instead of raw data) is to filter out fast variability that plays the role of noisy fluctuations. It will be interesting, in future work, to analyze how such stochastic factors affect the community structure obtained.

Thus, for each 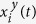, where *x* indicates either SAT or GH, *i* indicates the geographical location, *y* indicates the year and *t* indicates the month within that year, we first compute the seasonal cycle as 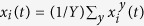 where *Y* is the number of years (64 or 65 depending on the month). Then, for each pair of time-series, *i* and *j*, we compute the lagged cross-correlation of the seasonal cycles, 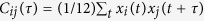, and determine their mutual lag, 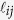, as the value of *τ* that maximizes *C*_*ij*_(*τ*). The seasonal cycle is by definition periodic, therefore, we search for a maximum in *τ* ∈ [0, 11]^39^,^40^. With 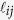, we calculate 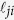 as: 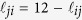 if 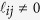, else 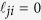. It is worthwhile to remark that correlation analysis is not used for determining the strength of the link between the two geographical locations: the actual value of 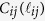 is disregarded, and only the value of 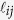 is used, to find regions with the same lag.

If the mutual lags among any three regions (*i*, *j*, *k*) are well defined, they should satisfy:


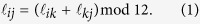


To fix the ideas, let us consider that *i* is a region in continental Europe, *j* is in the tropical eastern Pacific Ocean and *k* is in southern South America. If the lag between *i* and *j* is 8 months, and the lag between *i* and *k* is 6 months, then, the lag between *j* and *k* should be 2 months.

Therefore, one vector containing the lags between a region, *k*, and any other region, *i*, 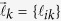, contains in fact all the information needed for computing the lag between any two regions *i* and *j*: if we know 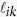 and 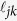, 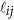 can be calculated from Eq. (1).

However, because we consider monthly-averaged data, 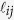, 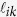 and 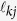 are integer numbers of months, and thus, because of round-off errors (the real lags are not necessarily integer numbers) Eq. (1) will not hold for all the triples (*i*, *j*, *k*).

In order to identify the regions that have well-defined lags among them, we chose a reference node *i*, and, for each other node *j*, we test Eq. (1) for all the possible *k*s. If the relation is satisfied in more than 50% of the cases, we consider 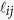 to be a well defined lag, otherwise no value is assigned. This is in fact a simple work-around solution to a complex optimisation problem: how to remove the minimum number of 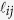 values, so that Eq.(1) is valid for all the remaining ones.

**Figure 1:**
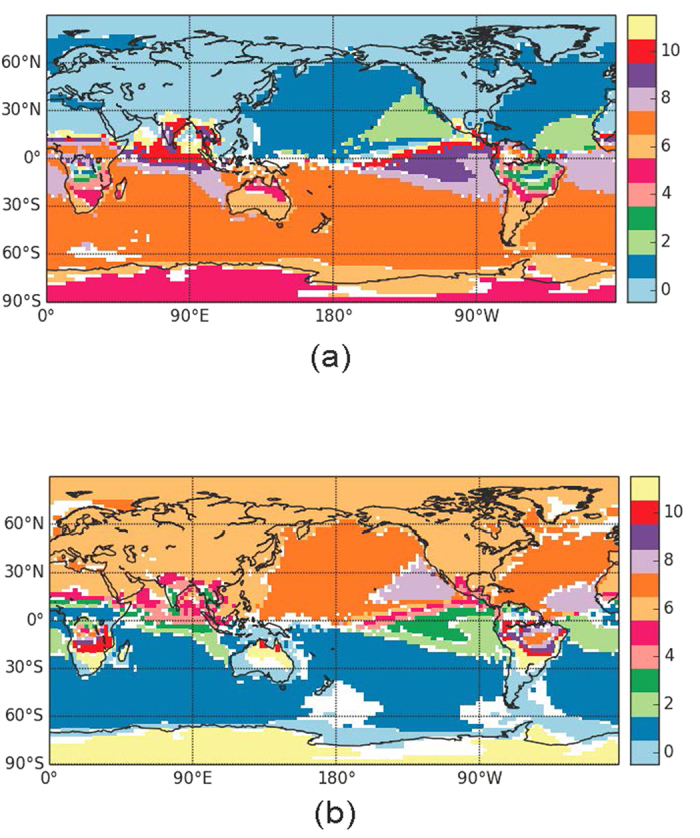
Communities obtained from computing mutual lags among SAT time-series. Regions depicted with the same color have a synchronous seasonal cycle, while the lag between two regions can be computed by subtracting the numbers associated with each color. Panel (a) was obtained by using a reference node located in continental Europe (Rome), while panel (b), a reference node in southern South America (Buenos Aires). The white areas indicate regions in which the lag with the reference point is not well-defined. Python 2.7 (https://www.python.org/) and the Basemap library (https://pypi.python.org/pypi/basemap/1.0.7) were used to create these maps.

Then, the information about all mutual lags, 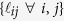, can be summarized in just one map, which displays the lags between a region, *k*, and any other region *i* (i.e., displays the vector 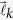), because, from this map, any lag 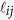 can be calculated using Eq.(1). For SAT time-series, the resulting map is plotted in Fig. 1a for a reference region in continental Europe (Rome) and in Fig. 1b for one in southern South America (Buenos Aires). In these plots, all the areas sharing the same color present a seasonal cycle in phase, and the white areas indicate regions in which the lag with the reference point is not well-defined. It is worthwhile to note that, while a precise characterization of the effect of the 50% coefficient for defining lag-times was not performed, it was indeed verified that the community structure was robust with respect to variations of this coefficient: an increased tolerance (i.e., a decrease of the number of cases that we require that Eq. (1) holds) only lead to a reduction of the white areas located at the boundaries between communities.

The two panels are very similar; the white areas are a little fraction of the total area, and they are located at the boundaries of well defined regions, thus confirming a coherent community decomposition. We can see that, in spite of the fact that the annual solar forcing is zonally symmetric, the maps of lag times are heterogeneous. In particular, wide ocean areas have a one-month delay with respect to the landmasses. In the eastern boundaries of the oceans this delay reaches two months and even three months in El Niño region. While the one-month delay can be expected due to thermal inertia of the water respect to the land, the longer delays have no straightforward explanation and require further investigation. A comparison of Fig. 1a,b confirms that the ‘transitivity’ property described by Eq. (1) holds: as a simple example, let us consider three geographical regions located in south Argentina, south Australia and Canada. Summer in south Australia occurs at the same time as winter in Canada, and winter in Canada occurs at the same time as summer in south Argentina; thus, south Argentina and Australia are expected to belong to the same climate community, because in south Argentina and Australia summers and winters occur at the same time. The community that contains south Argentina and Australia is expected to have a lag-time of six months with respect to the community that includes the continental landmasses in the north hemisphere (where Canada is), and these facts are indeed observed in Fig. 1a,b.

**Figure 2:**
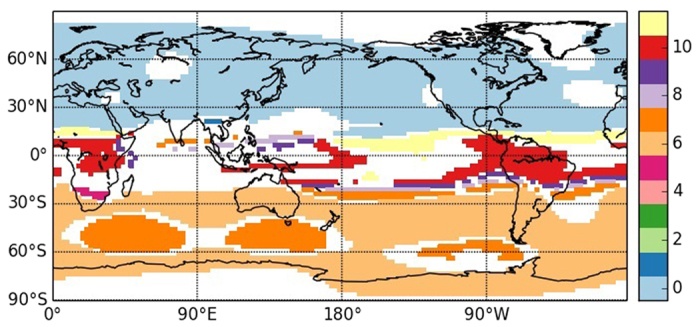
As Fig. 1a but computing the lag times from time-series of geopotential height at 500 hPa. Python 2.7 (https://www.python.org/) and the Basemap library (https://pypi.python.org/pypi/basemap/1.0.7) were used to create this map.

By applying this methodology to the geopotential height at 500 hPa, we uncover a very different community structure, displayed Fig. 2. In this case, due to the fact that the seasonal cycle is highly non-linear and heterogeneous, the white areas with not well-defined lags increase with respect to the SAT lag map. In particular, a wide part of the equatorial belt, as well as the polar region, have undefined lags. Also, in the northern hemisphere, two regions with undefined lags are consistent with the North Atlantic Oscillation pattern, which on long time-scales can act as a source of noise for the lag determination. Nevertheless, several consistent features can be seen, including the six-month symmetry between the two hemispheres.

## Symbolic Method

The second method proposed for community identification allows to uncover regions that share similar symbolic patterns of climate variability. To rule out similarities which are due to the periodicity induced by the solar annual cycle, the analysis is now performed on *anomaly* time-series, *y*_*i*_(*t*), computed by subtracting the seasonal cycle to the raw data. As in ref. 35, we first remove the fast anomaly fluctuations by using a one-year running mean.

In order to construct a network in which regions with similar climate are connected, we first use symbolic analysis and transform each time-series, *y*_*i*_(*t*), in a symbolic sequence, *s*_*i*_(*t*). Next, for each symbolic sequence we calculate the transition probabilities, *M*_*i*_(*α*, *β*), among all possible pairs of symbols, *α* and *β*. Specifically, we compute the number of times *β* occurs after *α*, over the total number of transitions. The transition probabilities (TPs) describe the statistics of the symbolic sequence. In order that two regions, *i* and *j*, with similar (dissimilar) TPs, are strongly (weakly) linked, we define the weight of the link between *i* and *j* as


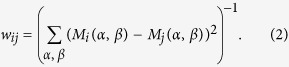


Next, we construct a network by considering only the strongest links, *i.e.*, we threshold {*w*_*ij*_} and obtain the adjacency matrix, *A*_*ij*_ = H(*w*_*ij*_ − *W*), where *H* is the Heaviside step-function and *W* is a threshold chosen such that each node is connected, on average, to 5% of the Earth surface (see the Supplementary Information, SI, for a discussion of the role of *W*). Then, we apply the Infomap algorithm of community identification^7^,^35^. In the SI we also demonstrate the robustness of the results by presenting the communities detected by different algorithms. To summarize, in this second method, the symbolic information obtained from *N* time-series is encoded in *N* TP matrices, and then we identify the regions which have similar TPs.

There are many ways of perform the symbolic data reduction. Here we use the method of *ordinal analysis*^41^,^42^,^43^ because it has been proven useful to construct climate networks^23^,^27^,^39^ (a comparison with other symbolic methods is presented in the Supplementary Information, SI). In this approach, each time series is divided into non-overlapping segments of length *Q*, and each segment is assigned a symbol, *s*, (known as ordinal pattern) according to the ranking of the values inside the segment. For example, with *Q* = 3, if *y*_*i*_(*t*) < *y*_*i*_(*t* + 1) < *y*_*i*_(*t* + 2), *s*_*i*_(*t*) is “012”, if *y*_*i*_(*t*) > *y*_*i*_(*t* + 1) > *y*_*i*_(*t* + 2), *s*_*i*_(*t*) is “210”, and so forth. Thus, the symbols take into account the *relative temporal ordering* of the values and not the values themselves. In this way, each symbol encodes information about the evolution of the time-series during *Q* months. In order to estimate the TPs with good statistics, the length of the time-series must be much longer than the number of possible transitions, *i.e., T* ≫ *Q*!^2^. Thus, with *T* = 773 months, we use *Q* = 3.

**Figure 3:**
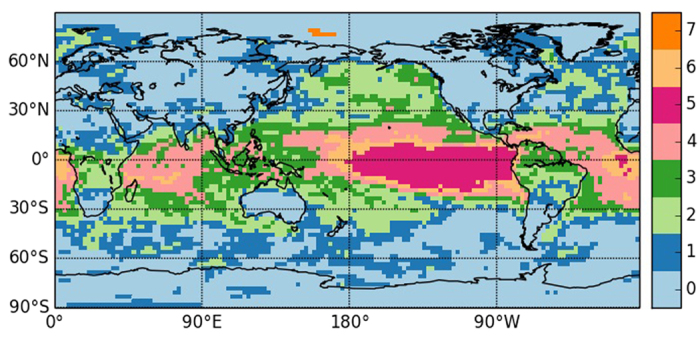
Communities obtained from the symbolic analysis of SAT anomalies. Regions depicted with the same color belong to the same community. Four macro-communities are identified: extratropical continents and oceans, tropical oceans and El Niño basin. Python 2.7 (https://www.python.org/) and the Basemap library (https://pypi.python.org/pypi/basemap/1.0.7) were used to create this map.

The community structure inferred from SAT anomalies is presented in Fig. 3. As it can be seen, the algorithm divides the world in 8 areas, labeled with different colors. These areas share similar dynamics, in the sense of similar symbolic transition probabilities. The continents in the two hemispheres are in the same community and a large coherent area is detected in the ENSO basin, while the oceans are divided in tropical and extratropical. A detailed analysis of these communities is provided in the SI.

It is important to remark that such community structure can not be inferred from networks that are constructed from correlation analysis (by using Pearson coefficient or mutual information). As our goal is that regions with similar climate belong to the same community, the classic tools are not useful, because they would not provide direct connections among extratropical regions. In order to belong to the same community two nodes must be part of the same group of strongly interconnected nodes, and in the correlation approach, where the links are prominently local, direct teleconnections across hemispheres are scarce (see SI for more details).

It is interesting to compare how these communities are related to those found in Fig. 1 through the seasonal cycle. There are borders among different communities that are indeed shared by the two sets, such as the extra-tropical coastlines, or the separation of northern from southern Australia and of southern South America from the rest of the continent.

The Infomap algorithm automatically converges towards a certain number of communities that cannot be directly controlled, as they are defined by the network structure. The number of communities depends on the network density, which is in turn modified by the threshold *W* used to construct the network. Increasing the network density makes the network to look like a giant coherent cluster, and the Infomap algorithm will detect a smaller number of communities. Decreasing the density will break the network in many small parts, and Infomap will detect them as many separate communities (see SI for details).

**Figure 4:**
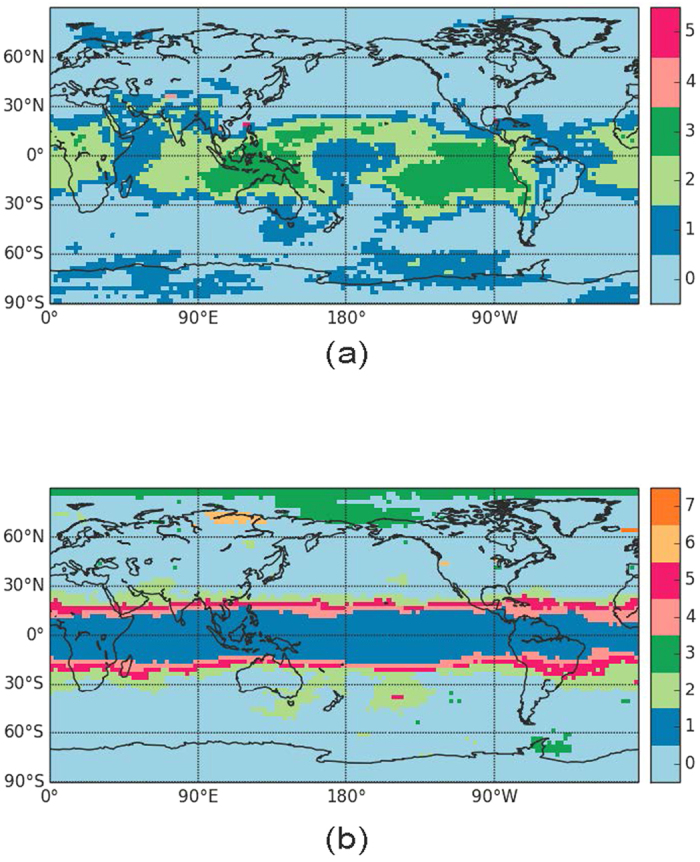
As Fig. 3 but for geopotential height anomalies at 1000 hPa (**a**) and 300 hPa (**b**). Python 2.7 (https://www.python.org/) and the Basemap library (https://pypi.python.org/pypi/basemap/1.0.7) were used to create these maps.

Figure 4 displays the communities extracted for GH anomalies at 1000 and 300 hPa. As it can be seen, increasing the height of the field implies a more zonal distribution of the communities: at 300 hPa the tropics form a belt that differentiates from the extratropical areas, which belong to the same community, the two are separated by strip-like communities, probably a signature of the subtropical jet. At 1000 hPa, instead, the effect of tropical convection is dominant, separating the low latitudes in two areas, the Maritime Continent together with the ENSO basin (perhaps a signature of the Walker circulation), and the rest of the tropics. The extratropics instead are grouped in the same community, regardless of the presence of landmasses.

## Community Analysis

In this section we analyse the statistical features of the communities uncovered with the symbolic method. In Fig. 3 we identified four macro-communities: extratropical continents (0) and oceans (2), tropical oceans (4) and ENSO basin (5). Then, there are also two boundary communities, 1 and 6, that are placed at the communities interfaces. Community 3, instead, includes precise areas (maritime continent, subtropical South American Monsoon system, stationary wave patterns of the North Pacific) although the connection among them is unclear. The remaining small community, 7, is clearly an artifact, and won’t be examined in the following analysis.

To test the goodness of the community decomposition, we checked which is the relative intensity of the internal connections within the communities in relation with the cross-community connections. In fact, for the decomposition to be meaningful, the communities must represent well connected regions, with weaker connections among them. To investigate this feature we computed the PDFs of the weights of the internal connections of each pair of communities, and we compared them to the PDF of the cross-community connections.

**Figure 5:**
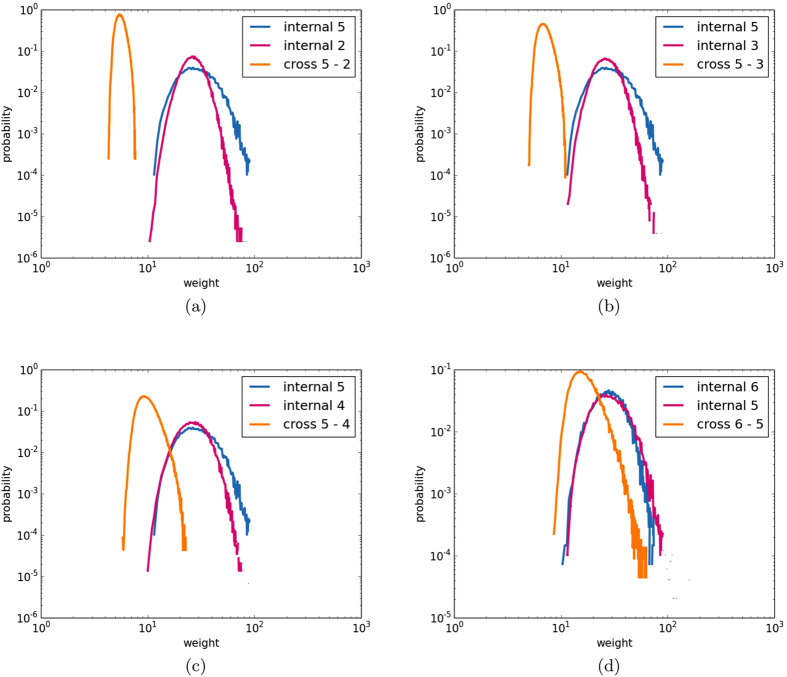
PDFs of the internal and cross-community weights for community 5 (El-Niño Basin) and other four communities (2, 3, 4 and 6, respectively panels (**a**–**d**)).

As an example, in Fig. 5 we report the PDFs of the internal links of community 5 together with the cross links between community 5 and communities 2, 3, 4 and 6. We also report the internal weights of these four communities. For geographically separated communities (e.g. 5 and 2) the PDFs are clearly separated, but, the more the communities tend to be geographically close, the more the cross-links PDF overlaps with the internal ones. However there is always a certain separation among the internal and cross-links PDFs, suggesting that the decomposition is meaningful even in the case of communities 5 and 6.

**Figure 6:**
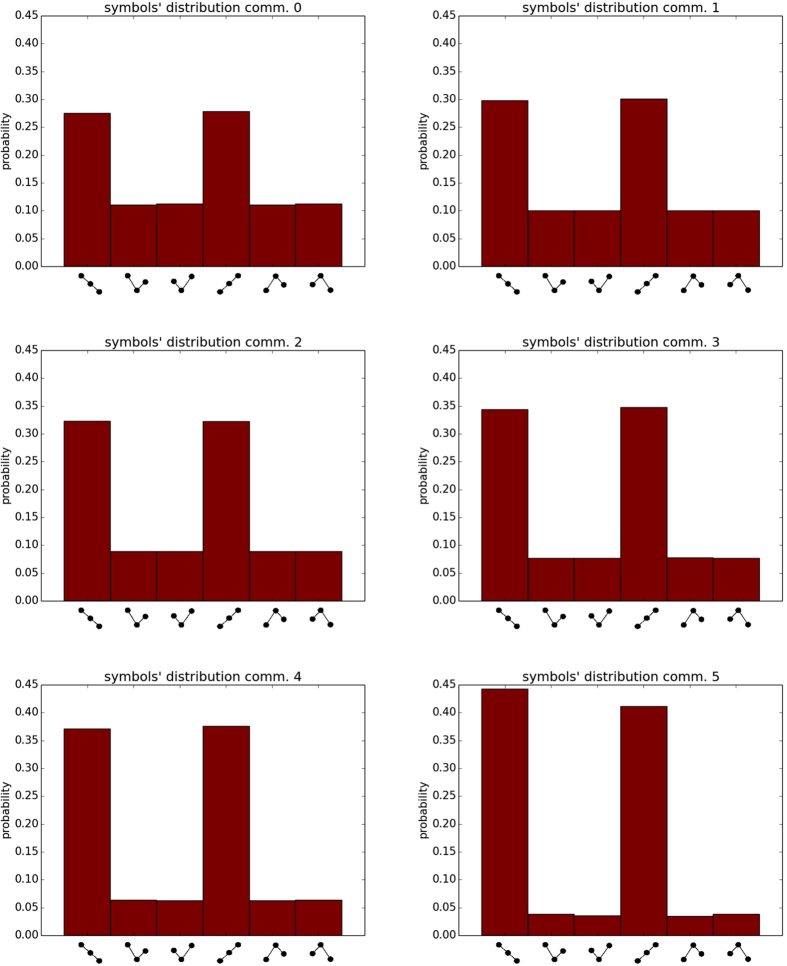
Average probabilities of symbol occurrence for the largest communities (0–5).

**Figure 7:**
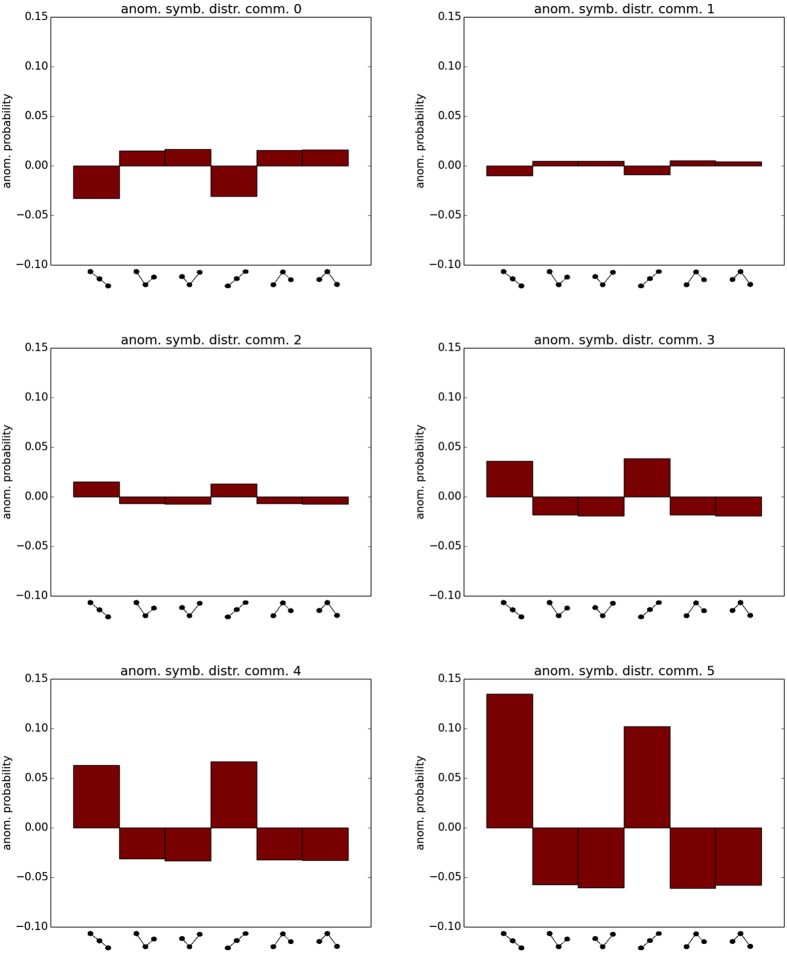
Anomalous symbols’ distributions. The bars indicate the differences between the probabilities displayed in Fig. 6 and the global probabilities (computed from all the nodes, without classifying them in communities).

We also analysed the symbolic dynamics of the nodes belonging to the same community. In Fig. 6 we report the average probabilities of symbol occurrence for the largest communities (0–5). As it can be seen the most prominent feature of these distributions is the presence of high probabilities in the “trend” patterns, that is those ordinal patterns (OPs) in which the data values either increase or decrease for two consecutive months. This characteristic is due to the application of the running mean to the time-series at the beginning of the analysis. To understand which are the features of the symbolic dynamics that are shared by the nodes of each community, we subtract to each histogram the global symbols’ distribution (that is computed from all the nodes, without classifying them in communities), obtaining the results presented in Fig. 7. From these new histograms is evident that, while equatorial communities (4, 5) have more pronounced trends, in the extratropical ones (0, 1) V-like or Λ-like symbols are more likely to occur. These features are in good agreement with the fact that autocorrelations are higher in the tropics with respect to the extratropics.

**Figure 8:**
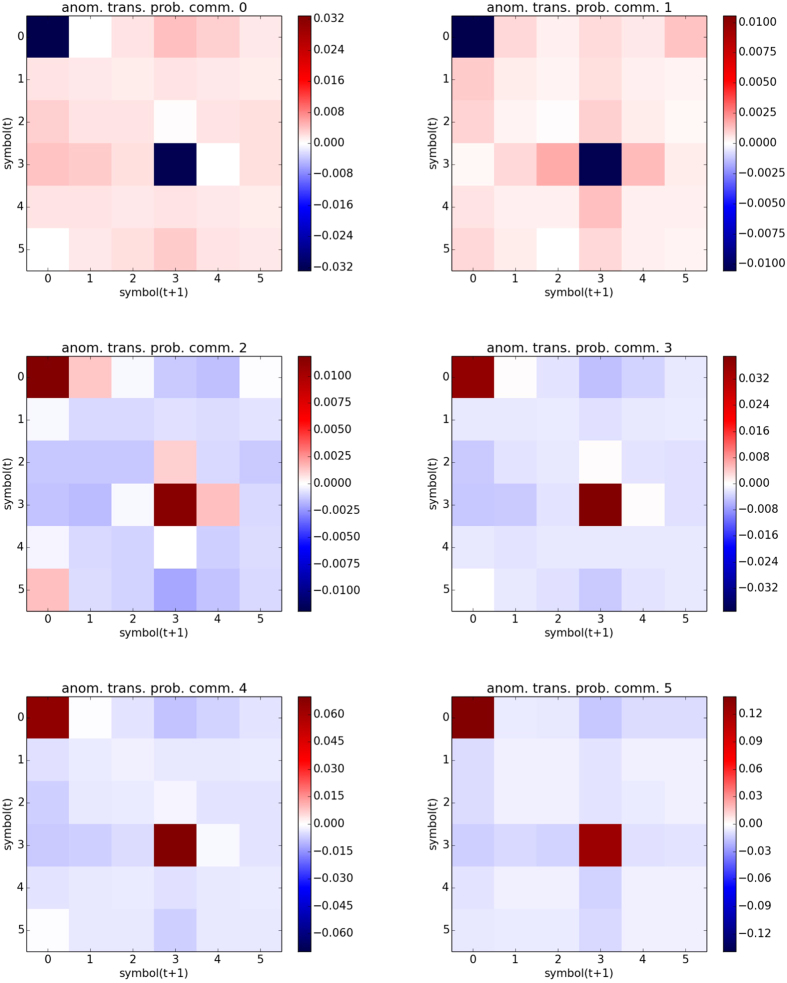
Anomalous transition probabilities. The color code indicates the difference between the average transition probabilities in each community and the global transition probabilities (computed from all the nodes, without classifying them in communities).

Lastly we analyse the average transition matrix for each community. Given the abundance of trend symbols the highest transition probabilities will be among them. However, since the connections are defined by the differences between the matrix elements, this common bias is removed by the subtraction in Eq. 3. To display more clearly the differences among the average transition matrices of the different communities, we repeat the procedure done for Fig. 7 and subtract the global transition matrix, obtaining the results presented in Fig. 8. In this figure we can see that in communities 0 and 1 there is an anomalous presence of transitions among the V-like and Λ-like patterns (labelled as 1, 2, 4 and 5) although the highest positive signal is among these patterns and trends (labeled 0 and 3). In the other communities the situation is the opposite, with an anomalous prevalence of transitions among the trends. These are reflected in the histograms of Fig. 5, where the cross-links among equatorial and extratropical communities show small weights (due to large distances between the transition matrices).

## Discussion

We have presented two methods to identify communities in dynamical complex systems using the properties of observed time-series. We tested the methods with climate data (surface air temperature and the geopotential height at two pressure levels), and uncovered communities that are consistent with main large-scale climate phenomena. The first method, based on computing mutual lags among the time-series through correlation analysis, uncovered communities formed by geographical regions with synchronous seasonal cycles. The second method, based on symbolic analysis, identified communities formed by geographical regions where the climate variability displays similar symbolic patterns.

The proposed methods allow analyzing complementary properties of our climate. The first one uncovers regions with in-phase seasonal cycle, and thus, it is appropriated for characterising spatiotemporal patterns of seasonality, and can even provide new insight on their evolution. The second method for community detection uncovers regions with similar statistical properties of climate temporal variability. In the Supplementary Information we analyse the influence of the threshold *W* and use three other community detection algorithms, to stress the significance and robustness of the uncovered communities. The proposed methods could be used for analyzing local statistics, detecting regime transitions, performing model inter-comparisons, etc. Other applications include the analysis of specific geographical regions, to uncover sub-areas with similar micro-climate. Exploiting the approach of interactive and multi-layer networks^10^,^24^,^26^,^35^, these methods could also be valuable for studying large scale circulation dynamics.

These two methods can be used to analyse other real-world, dynamical complex systems. A relevant issue is that many of these systems are represented by nodes of different sizes, as in climate networks. Here, for the sake of clarity, we have not taken into account the fact that the geographical regions have different sizes, but this could be taken into account by using a similar approach as in ref. 22. Because both methods were demonstrated with short and noisy datasets, they can be used for analysing brain signals, to uncover brain regions with in-phase dynamics or with similar symbolic dynamics.

## Additional Information

**How to cite this article**: Tirabassi, G. and Masoller, C. Unravelling the community structure of the climate system by using lags and symbolic time-series analysis. *Sci. Rep.* **6**, 29804; doi: 10.1038/srep29804 (2016).

## Supplementary Material

Supplementary Information
