# Supplementary material for: Unravelling the community structure of the climate system by using lags and symbolic time-series analysis
Source: Sci Rep. 2016 Jul 11;6:29804. doi: 10.1038/srep29804 (PMC4942694; doi:10.1038/srep29804)
Supplement: Supplementary Information [file srep29804-s1.pdf]

Supporting Information for ”Unravelling the community structure of the climate system by using lags and symbolic analysis”

Giulio Tirabassi[1] **and Cristina Masoller**[1,*]

[1]Departament de Fisica, Universitat Politecnica de Catalunya, Colom 11, ES-08222 Terrassa, Barcelona, Spain
[*]cristina.masoller@upc.edu

ABSTRACT

Here we present an additional analysis of the communities uncovered with the symbolic method proposed in the main manuscript: we discuss the role of the threshold, we demonstrate the robustness of the communities found by using other algorithms, and we compare with the community structure when the network is built with correlation analysis.

Comparison with other community detection algorithms

In the main manuscript the Infomap algorithm was used to identify the communities. Infomap was chosen for two reasons. First, it has been employed in previous climate network studies, such as[1], and second, in our study it provided a community structure that was less noisy than the other algorithms tested. As an example we report in Fig. 1 the comparison among the community structures obtained using Infomap and other three popular community detection algorithms.

In first approximation, the methods produce indeed comparable results, demonstrating the robustness of the structures obtained; however the Infomap decomposition seems to be less noisy.

Influence of the symbolic representation

The community identification algorithm uses a symbolic representation of time-series, known as ordinal analysis, by which SAT anomalies are represented by a set of ordinal patterns. In this way the information of the evolution of the SAT anomalies at the seasonal time-scale is encoded into transition probability matrices. This procedure is quite general, and can be applied to any symbolic representation of the time-series. To study the influence of the symbolic encoding method, here we use an alternative approach and digitalise the time-series with a finite set of values.

We performed this analysis using 10 equally spaced values, ranging from the maximum to the minimum value of each time-series, then we followed the same procedure as in the main text: we computed the transition matrices, and their distances, and by thresholding the distances we obtained a climate network; finally the Infomap algorithm was used to identify the communities. The results are shown in Fig. 2.

As it can be seen, the community structure obtained with this symbolic representation is much noisier than the one obtained through ordinal patterns. This is probably due to the fact that, with the ordinal patterns, each symbol encodes information about the *seasonal evolution* of SAT anomalies; in contrast, with the simpler symbolic encoding used here, the symbols only encode information about the coarse grained SAT anomaly values. We speculate that by using “blocks” composed by several coarse grained values[2], a less noisy community structure could be revealed, and this will be an interesting issue to investigate in future work.

Influence of the threshold used to construct the network

In order to construct a climate network, the weights matrix has to be pruned by using an adequate threshold W. Decreasing the threshold leads to a more connected network, while increasing it results in a sparser one. The number of communities depends on the number of connections, which in turn depends on the threshold. In order to uncover a coherent, well-defined community structure, the threshold has to be carefully chosen.

We report in Fig. 3 the number of communities and the average degree as a function of the threshold. It can be seen that there is a negative correlation between the number of communities and the average degree. The fragmentation of the network into smaller communities (as community 7 in Fig. 1a or communities 8, 9 and 10 in Fig. 1b) could be due to the removal of relevant links that keep the bigger communities together. Thus, to obtain a meaningful community structure, we selected a

threshold that provided the best compromise between the need to limit the small-communities-proliferation and the need to include in the network only the relevant links.

We stress that the same qualitative behaviour is found also when using other community detection algorithms.

Comparison with networks constructed by using the Pearson correlation coefficient

In this section we contrast the community structure obtained by using the proposed methodology (based on transition probabilities computed with symbolic analysis), with that obtained with the classical approach, which measures the dynamical similarity of two time series with the Pearson correlation coefficient, r_{ij}. As in[3] we use a threshold $W = 0.5$ to prune the r_{ij} matrix.

Applying the Infomap algorithm to the obtained network results in 8604 communities, but only 20 are composed by more than 2 nodes. Figure 4 shows the largest 16 communities.

As it can be seen, only communities 0 and 1 correspond to coherent structures, namely El-Niño basin, and the tropical oceans, while the others appear to be just noise.

We also report the effect of the threshold value over the community structure in this case. As it can be seen in Fig. 5 the number of communities increases as the threshold is increased, as it occurs when using ordinal analysis (Fig. 3); however, here the change is more abrupt (note the logarithmic vertical scale), and it occurs at low values of the threshold.

Moreover, when a low threshold is used in order to limit the number of communities, the community structure obtained is meaningless, as it can be seen in Fig. 6.

References

1. Tantet A. & Dijkstra, H.A. An interaction network perspective on the relation between patterns of sea surface temperature variability and global mean surface temperature. *Earth Syst. Dynam.* **5**, 1 (2014).
2. Barreiro, M., Marti, A.C. & Masoller, C. Inferring long memory processes in the climate network via ordinal pattern analysis. *Chaos* **21**, 013101 (2011).
3. Tsonis A.A. & Roebber, P.J. The architecture of the climate network. *Physica A* **333**, 497-504 (2004).
4. Newman, M.E.J. Finding community structure in networks using the eigenvectors of matrices. *Phys. Rev. E 74*, 036104 (2006).
5. Clauset, A., Newman, M.E.J. & Moore, C. Finding community structure in very large networks. *Phys. Rev. E* **70**, 066111 (2004).
6. Pons, P. & Latapy, M. Computing communities in large networks using random walks. *J. Graph Algorithms Appl.* **10**, 191-218 (2006).

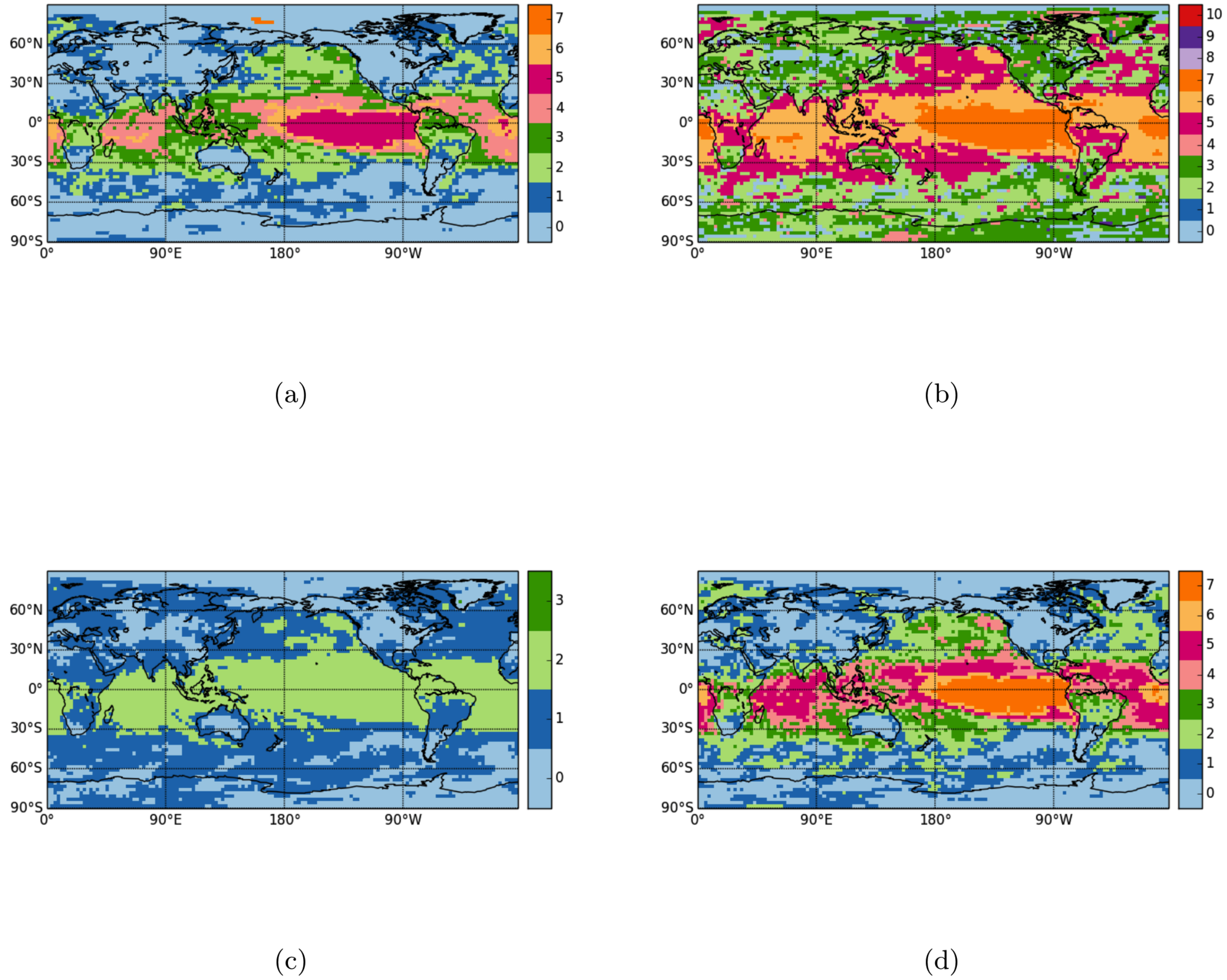

Figure 1. Community structure found by analyzing the same network with four popular community detection algorithms. **(a)** Infomap (same as Fig. 3 in the main text); **(b)** Leading Eigenvector[4] (threshold used: 35); **(c)** Fast Greedy Modularity Maximization[5] (threshold used: 35); **(d)** Walktrap[6]. Python 2.7 (https://www.python.org/) and the Basemap library (https://pypi.python.org/pypi/basemap/1.0.7) were used to create these maps.

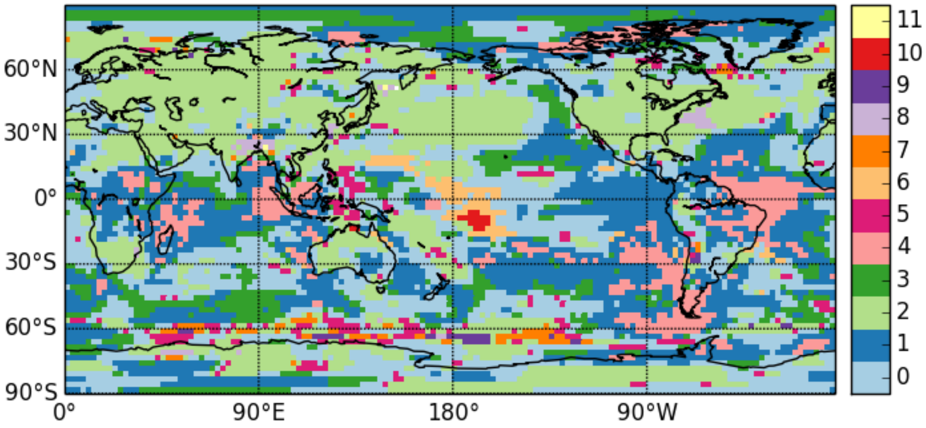

Figure 2. Community structure found with Infomap when the network is constructed by using a symbolic transformation that only encodes information about the coarse grained SAT anomaly values. Python 2.7 (https://www.python.org/) and the Basemap library (https://pypi.python.org/pypi/basemap/1.0.7) were used to create this map.

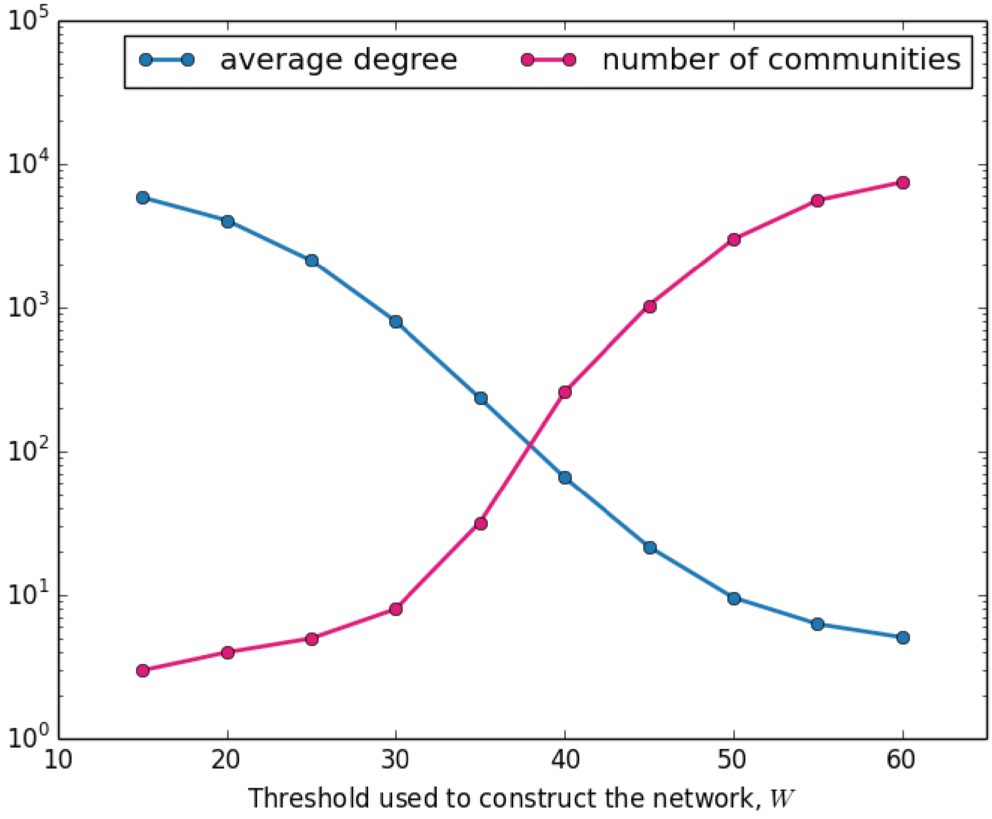

Figure 3. Dependence of the network average degree and of the number of communities with the value of the threshold used to construct the network, W. For the community structure shown in Fig. 1, the threshold used was $W = 30$.

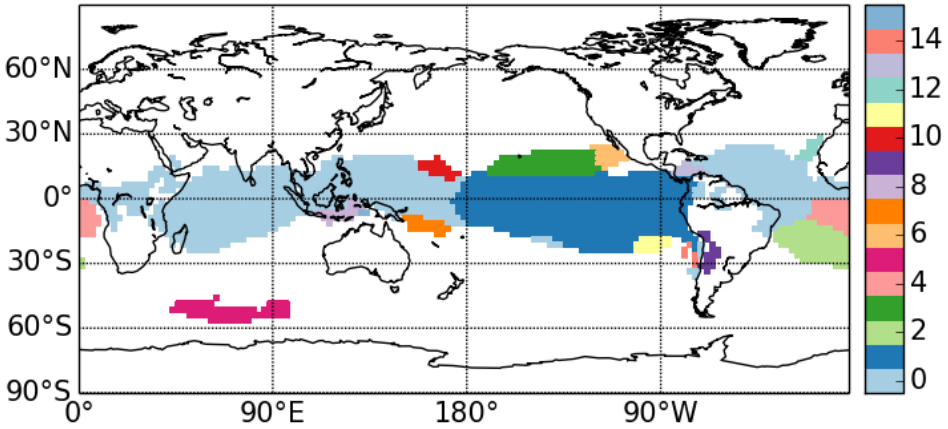

Figure 4. Community structure found with Infomap when the network is constructed by using the Pearson correlation coefficient as a measure of dynamical similarity. The value of the threshold used to construct the network is $W = 0.5$. For clarity, only the largest 16 communities are shown. Python 2.7 (https://www.python.org/) and the Basemap library (https://pypi.python.org/pypi/basemap/1.0.7) were used to create this map.

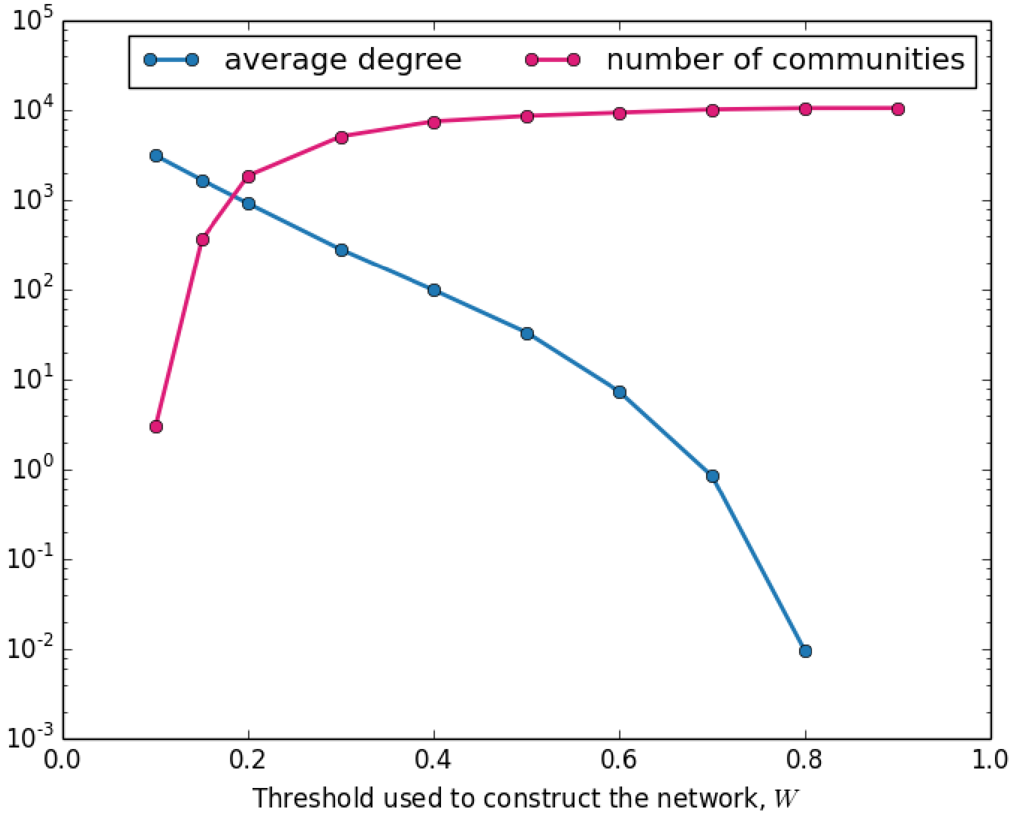

Figure 5. Dependence of the network average degree and of the number of Infomap communities from the threshold value for a network obtained thresholding the covariance matrix of the data.

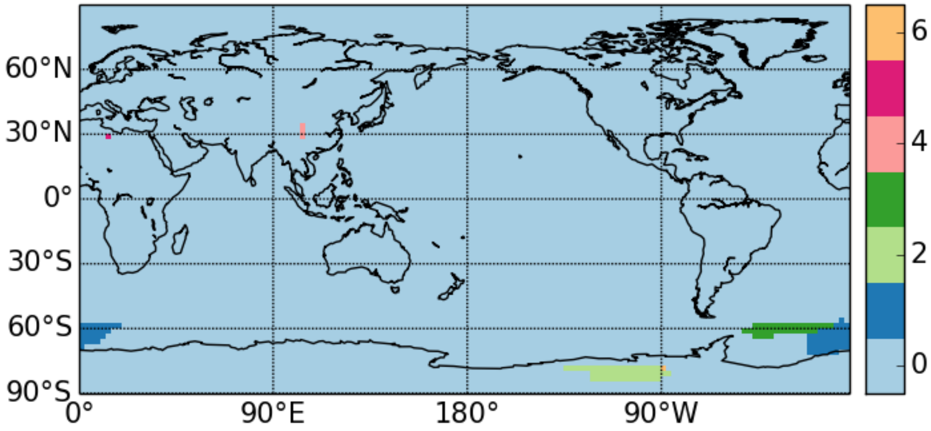

Figure 6. As Fig.4 but with $W = 0.107$. Python 2.7 (https://www.python.org/) and the Basemap library (https://pypi.python.org/pypi/basemap/1.0.7) were used to create this map.
